# Nef-mediated enhancement of cellular activation and human immunodeficiency virus type 1 replication in primary T cells is dependent on association with p21-activated kinase 2

**DOI:** 10.1186/1742-4690-8-64

**Published:** 2011-08-05

**Authors:** Kevin C Olivieri, Joya Mukerji, Dana Gabuzda

**Affiliations:** 1 Department of Cancer Immunology and AIDS, Dana-Farber Cancer Institute, Boston, MA, USA; 2 Department of Neurology, Harvard Medical School, Boston, MA, USA

## Abstract

**Background:**

The HIV-1 accessory protein Nef is an important determinant of lentiviral pathogenicity that contributes to disease progression by enhancing viral replication and other poorly understood mechanisms. Nef mediates diverse functions including downmodulation of cell surface CD4 and MHC Class I, enhancement of viral infectivity, and enhancement of T cell activation. Nef interacts with a multiprotein signaling complex that includes Src family kinases, Vav1, CDC42, and activated PAK2 (p21-activated kinase 2). Although previous studies have attempted to identify a biological role for the Nef-PAK2 signaling complex, the importance of this complex and its constituent proteins in Nef function remains unclear.

**Results:**

Here, we show that Nef mutants defective for PAK2-association, but functional for CD4 and MHC Class I downmodulation and infectivity enhancement, are also defective for the ability to enhance viral replication in primary T cells that are infected and subsequently activated by sub-maximal stimuli (1 μg/ml PHA-P). In contrast, these Nef mutants had little or no effect on HIV-1 replication in T cells activated by stronger stimuli (2 μg/ml PHA-P or anti-CD3/CD28-coated beads). Viruses bearing wild-type Nefs, but not Nef mutants defective for PAK2 association, enhanced NFAT and IL2 receptor promoter activity in Jurkat cells. Moreover, expression of wild-type Nefs, but not mutant Nefs defective for PAK2 association, was sufficient to enhance responsiveness of primary CD4 and CD8 T cells to activating stimuli in Nef-expressing and bystander cells. siRNA knockdown of PAK2 in Jurkat cells reduced NFAT activation induced by anti-CD3/CD28 stimulation both in the presence and absence of Nef, and expression of a PAK2 dominant mutant inhibited Nef-mediated enhancement of CD25 expression.

**Conclusion:**

Nef-mediated enhancement of cellular activation and viral replication in primary T cells is dependent on PAK2 and on the strength of the activating stimuli, and correlates with the ability of Nef to associate with PAK2. PAK2 is likely to play a role in Nef-mediated enhancement of viral replication and immune activation *in vivo*.

## Introduction

The HIV-1 accessory protein Nef is an important determinant of lentiviral pathogenicity (reviewed in [1]). Infections with Nef-deleted strains of HIV-1 [2,3] or SIVmac [4,5] result in limited disease progression in humans and primates, respectively. The mechanisms by which Nef enhances viral replication and pathogenicity are unclear. A conserved feature of lentiviral *nef *genes is the ability to enhance viral replication in freshly isolated T cells that are infected and subsequently activated 2 to 5 days post-infection [6-11]. Under these conditions, Nef+ viruses replicate with faster kinetics and peak at higher levels (approximately 10-fold) than Nef- viruses. In contrast, Nef has little or no effect on viral replication when T cells are activated prior to infection [7]. In addition to enhancing viral replication in freshly isolated T cells, Nef mediates downregulation of cell surface receptors via interaction with the endocytic machinery. Downmodulation of cell surface CD4 reduces interference with viral envelope protein function [12,13]. Nef also downmodulates MHC Class I, which protects infected cells from CTL-mediated lysis [14,15]. Thus, Nef-mediated effects on viral replication and pathogenesis may depend in part on its ability to enhance viral replication in resting CD4+ T cells.

In resting T cells, HIV-1 viral replication is blocked at a step prior to integration [16]. This restriction is overcome when resting T cells are activated in response to TCR stimulation [16-18]. Nef, which is expressed early after infection in resting T cells [19], increases the number of T cells that activate NFAT and NF-κB promoter elements [20-23], secrete IL-2 [24], and express activation markers such as CD25 [25] and CD69 [26] in response to TCR stimulation. Nef appears to lower the threshold required for T cell activation, which may increase the permissiveness of cells for productive infection.

Previous studies suggest that Nef lowers the activation threshold by interacting with components of the T cell signaling machinery. Nef, via its SH3-binding P_72_xxP_75 _motif, associates with the Src Family kinases (SFKs) Fyn [27] and Lck [28,29], which are proximal signaling molecules activated immediately after TCR stimulation [30]. Nef also modulates the activation of downstream effectors important for activation-induced cytoskeletal rearrangement including PAK2, CDC42, Vav [31,32], WASP (Wiscott-Aldrich Syndrome protein) [33], and the Ezrin Radixin Moesin (ERM) proteins Merlin [34] and cofilin [35,36]. Nef associates with an activated form of PAK2 [37-39], a serine/threonine kinase important in T cell activation and stress responses, in a multiprotein complex found in detergent insoluble lipid rafts [40,41]. This association is dependent on both CDC42 and Vav1 and, possibly, β-PIX [42,43]. Functional links between SFKs and PAK2 through Vav1 and CDC42 suggest Nef-PAK2 association may serve as a marker for a Nef-multiprotein signaling complex capable of altering T cell responsiveness via interaction with multiple host cell factors. Despite extensive characterization of the molecular determinants of Nef-PAK2 association, the importance of this association for Nef function is still unclear.

The P_72_xxP_75 _motif of Nef is important for PAK2 and SFK-association and contributes to MHC Class I downmodulation [44,45]. Mutation of this motif also abrogates Nef-mediated enhancement of HIV-1 replication [46,47] and T cell activation [20]. It is therefore difficult to distinguish requirements for PAK2-association, SFK-association, and MHC Class I downmodulation in Nef-mediated enhancement of replication and T cell activation. We previously indentified residues important for PAK2 association, but dispensable for CD4 or MHC Class I downmodulation [48-50]. These determinants of PAK2 association are located in a hydrophobic binding surface formed by Clade B consensus positions 85, 89, 90, 186,187,188, and 191 [48]. Mutation of residue 191, however, disrupts Nef association with Vav [31] and SFKs [51]. Mutations at position 191 (F191H and F191R) do not abrogate Nef-mediated enhancement of NFAT activity in cells stimulated for 18 h with 1 μg/ml PHA-P [52]. However, these mutants are unlikely to be completely null for PAK2-association [48]. The role of Nef-PAK2 association in Nef-mediated enhancement of T cell activation and viral replication under various levels of cell stimulation remains to be determined. Therefore, further analyses of Nef variants bearing mutations in the hydrophobic binding surface may provide insight into the biological role of the Nef-PAK2 complex in Nef-mediated enhancement of viral replication and T cell activation.

Here, we demonstrate that HIV-1 Nef mutants defective for the ability to associate with PAK2 are also defective for the ability to enhance viral replication in freshly isolated primary T cells that are infected and subsequently activated by sub-maximal stimuli. Furthermore, these Nef mutants are defective for the ability to enhance responsiveness of Nef-expressing and bystander primary T cells to activation induced by sub-maximal stimuli. siRNA knockdown of PAK2 inhibited NFAT activation both in the presence and absence of Nef, and expression of a dominant-negative PAK2 mutant abrogated Nef-mediated enhancement of CD25 expression in Jurkat cells. These findings suggest a model in which Nef interacts with PAK2 to enhance the responsiveness of infected cells and bystander cells to activating stimuli. Thus, PAK2 is likely to be important for Nef-mediated enhancement of viral replication and immune activation in vivo.

## Results

### Nef association with activated PAK2 is not important for enhancement of viral infectivity

**Figure 1:**
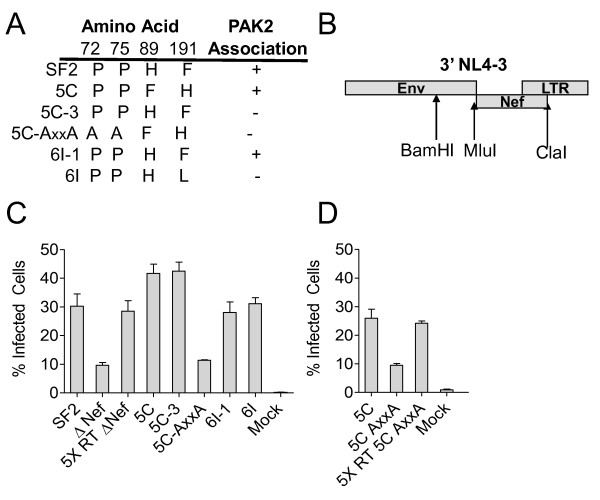
**The ability of Nef to associate with PAK2 is not important for enhancing viral infectivity**. **A**. Key residues in the amino acid sequence of Nef variants used in this study. **B**. Primary Nefs and mutant Nefs were inserted into a modified NL4-3 provirus via a BamHI site in Env and an artificial ClaI site in the viral LTR. SF2 Nef was inserted via an artificial MluI site at the start of Nef and the ClaI site. **C and D**. MAGI R5+ cells were incubated with 5,000 ^3^H cpm of RT activity of each viral variant for 6 h. At 36 h post-infection, the cells were fixed and stained for β-galactosidase activity. Average percent of infected (β-galactosidase+) cells from triplicate infections +/standard errors of the mean (SEM) are shown.

To determine if the ability of Nef to associate with PAK2 is important for its ability to enhance viral replication, we constructed a panel of NL4-3-based variants containing *nef *genes with known abilities to associate with PAK2. For these studies SF2 *nef *and the primary *nef *genes 5C and 6I [34,48], which have been extensively characterized for their ability to associate with PAK2, were inserted into the pNL4-3 provirus (Figure 1A and 1B). A single amino acid mutant of 5C, 5C-3, disrupts Nef-PAK2 association, but does not affect CD4 and MHC Class I downmodulation [48]. The 5C-A_72_xxA_75 _mutation in the PXXP motif disrupts SH3 binding and SFK association, PAK2 association, MHC Class I downmodulation, and virion infectivity [53]. This pleiotropic mutant was included because it was extensively characterized in previous studies, and has been shown to disrupt Nef-mediated enhancement of viral replication [54]. The primary *nef *gene 6I is defective for PAK2 association, and the 6I-1 mutant contains an L191F mutation and possesses wild-type ability to associate with activated PAK2 [48]. A ΔNef virus with a frameshift mutation in Nef introduced in an XhoI site within Nef (ΔXhoI) was used as a negative control [7,55].

Nef enhances virion infectivity in single round infection assays, even when the virus is produced in CD4-negative cells [56]. Contradictory data exist regarding whether or not abrogating the ability of Nef to associate with PAK2 affects virion infectivity [52,57]. To determine if Nef proteins defective for PAK2-assocation display reduced enhancement of infectivity during a single round of replication, we infected CD4+ CXCR4+MAGI cells, which express β-galactosidase under the control of the LTR, with equal amounts of virus normalized by RT activity. At 36 h post-infection, SF2 Nef, 5C, 5C-3, 6I, and 6I-1 viruses were 3- to 4-fold more infectious than the ΔNef and 5C-A_72_xxA_75 _viruses (Figure 1C). To achieve an equal MOI, a second infection was performed with 5-fold more RT units of the ΔNef or 5C-AxxA virus (Figure 1C and 1D). This dose resulted in equivalent infectivity between ΔNef virus and the initial dose of the SF2 Nef virus (Student's t test p = 0.7), or between 5C-AxxA virus and the initial dose of 5C virus (p = 0.5). Equivalent MOIs were therefore achieved using equal RT units of wild type and mutant Nef bearing virus and 5-fold more RT units of the ΔNef or 5C-AxxA virus. These data indicate that PAK2 association is not important for Nef-mediated enhancement of infectivity during a single round of viral replication.

### Nef-mediated enhancement of viral replication is highly dependent on the strength of activating stimuli

**Figure 2:**
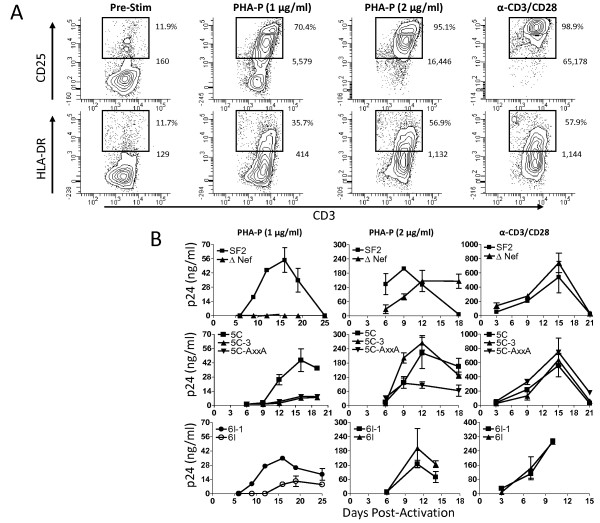
**Nef-mediated enhancement of replication is highly dependent on the strength of activating stimuli and the level of T cell activation**. **A**. Freshly-isolated PBMC were cultured in the presence of IL-2 (10 U/ml) alone or with either PHA-P (1 μg/ml), PHA-P (2 μg/ml), or α-CD3/CD28 beads (1 bead per cell) for three days. On day 3, cells were stained with α-CD3-FITC and α-CD25-PE or α-CD3-FITC and α-HLA-DR-PE or isotypic controls and analyzed by flow cytometry. %CD25+, %HLA-DR+, and CD25 and HLA-DR median fluorescence intensity of the CD3+ population is shown. Results are typical of three donors. **B**. Freshly isolated PBMC were infected with an equivalent MOI. Three days post-infection cells were activated with IL-2 (10 U/ml) and either PHA-P (1 μg/ml), PHA-P (2 μg/ml), or α-CD3/CD28 beads (1 bead per cell) for three days. Three days post-activation, the stimulation media was removed and replaced with IL-2 (10 U/ml)-containing media. p24 in culture supernatants was monitored by ELISA.

Although Nef expression increases the percentage of T cells responding to activating stimuli, the levels of activation markers or activation-dependent transcription are similar between Nef+ and Nef- cells [22,24]. Therefore, Nef appears to reduce the threshold of stimulation required for T cell activation [22,24]. To examine the relationship between Nef-PAK2 association and HIV replication in PBMC, we first sought to identify a sub-maximal stimulus that induced measurable T cell activation and sustained viral replication, but did not activate all cells. Previous reports describing effects of Nef on HIV-1 replication used 3 days of stimulation with PHA-P (1 μg/ml) [7], PHA-P (2 μg/ml) [6], or α-CD3/CD28-coated beads [19] in the presence of IL-2 to stimulate T cells or PBMC that were infected immediately after isolation. Each of these stimuli was evaluated for their ability to influence Nef-mediated enhancement of replication. IL-2 alone was also tested for the ability to activate freshly isolated PBMC. Cultures were stained for cell surface expression of CD3 and the activation markers CD25 and HLA-DR. Median fluorescence intensity (MFI) was calculated to define populations that contain multiple peaks of CD25 and HLA-DR expression within the CD3+ population. Prior to activation, PBMC cultures contained 8.3% CD25+ T cells (MFI 170) (data not shown). On day 3 post-activation, cultures with IL-2 alone contained 11.9% CD25+ cells (MFI 160) and 11.7% HLA-DR+ T cells (MFI 129). CD25- and HLA-DR-positive cells were more frequent in PBMC cultures stimulated with α-CD3/CD28-coated beads than in cultures stimulated with either dose of PHA-P (Figure 2A). Cultures stimulated with 1 μg/ml PHA-P contained the lowest percentage of CD25+ (70.4%) and HLA-DR+ (35.7%) cells. Cultures stimulated with 2 μg/ml PHA-P contained a similar frequency of CD25+ and HLA-DR+ cells compared to cultures stimulated with α-CD3/CD28 (95.1% CD25+ and 56.9% HLA-DR+ versus 98.9% CD25+ and 57.9% HLA-DR+, respectively). CD25 MFI was approximately 3-fold lower in cultures stimulated with 1 μg/ml PHA-P (MFI 5,579) than in cultures stimulated with 2 μg/ml PHA-P (MFI 16,446), and 12-fold lower than in cultures stimulated with α-CD3/CD28-coated beads (MFI 65,178). HLA-DR MFI was 3-fold lower in cultures stimulated with 1 μg/ml PHA-P (MFI 414) than cultures stimulated with 2 μg/ml PHA-P (MFI 1,132). In contrast to CD25 MFI, HLA-DR MFI was only 3-fold lower in cultures stimulated with 1 μg/ml PHA-P than in cultures stimulated with α-CD3/CD28-coated beads (MFI 1,144) and was similar between cultures stimulated with 2 μg/ml PHA-P and α-CD3/CD28-coated beads. CD25 is an early activation marker that is expressed at high levels 3 days post-activation; HLA-DR is upregulated at later time points [58]. CD25 MFI changed much more dynamically than % CD25+ when comparing stimuli of different strengths, in part reflecting changes in a CD25+ sub-population that expresses high levels of CD25. Thus, these stimuli provide three different levels of cellular activation, reflected by robust differences in CD25 MFI, which can be used to determine appropriate experimental conditions for measuring the effect of Nef on HIV-1 replication.

To examine whether Nef-PAK2 association is important for Nef-mediated enhancement of viral replication in primary T cells, we then tested a panel of viruses for their ability to replicate in freshly isolated PBMC under each of the conditions described above. We used each of these stimuli in the presence of IL-2 to activate freshly isolated PBMC that were previously infected with an equivalent infectious dose of virus. Viral replication was monitored by p24 ELISA of culture supernatants. In cultures stimulated with α-CD3/CD28 beads, viruses expressing Nef proteins capable of associating with activated PAK2 (SF2, 5C, and 6I-1) replicated at similar levels compared to viruses expressing Nef proteins defective for PAK2 association (5C-3, 5C-AxxA, and 6I) or ΔNef virus (Figure 2B). At this strong level of stimulation, there was no difference in levels of Nef+ and Nef- HIV-1 replication. When cultures were stimulated with 2 μg/ml PHA-P, ΔNef virus replicated more slowly than SF2 Nef virus, achieving peak levels of viral replication 3 days later. Under this condition, wild-type virus replicated to peak values that were 3-fold lower compared to those seen when cultures were stimulated with α-CD3/CD28 beads, and the 5C-AxxA virus replicated to 2-fold lower peak values compared to the 5C and 5C-3 viruses. No difference was observed between the 5C and 5C-3 viruses, or the 6I-1 and 6I viruses. At this intermediate level of stimulation, differences between Nef+ and Nef- viruses were detected but Nef-mediated enhancement of viral replication was considerably less than the 10-fold effect previously reported [7,9]. When 1 μg/ml PHA-P was used, wild-type virus replicated to a peak value 3-fold lower than the levels observed when 2 μg/ml PHA-P was used as a stimulus. Under this condition, the ΔNef virus failed to replicate to detectable levels (Figure 2B). CD25 MFI correlated positively with peak p24 levels of wild-type SF2 virus (Additional File 1, Figure S1A, p = 0.015, r = 0.898; Spearman correlation), and negatively with Nef-mediated enhancement of replication (Additional File 2, Figure S1B, p = 0.016, r = -0.892). In contrast, HLA-DR MFI did not correlate with viral replication (p = 0.44) or Nef-mediated enhancement of replication (p = 0.67). Therefore, increased CD25 MFI after 3 days of stimulation, rather than the % of CD25+ cells, was the best predictor of peak levels of viral replication and the ability of Nef to enhance viral replication.

### The ability of Nef to associate with activated PAK2 correlates with the ability to enhance HIV-1 replication in freshly isolated PBMC

The low level of activation following stimulation with 1 μg/ml PHA-P (Figure 2B) provides an optimal window to detect Nef-mediated enhancement of HIV-1 replication in freshly isolated PBMC. Under these experimental conditions, the 5C virus replicated to peak levels ~5-fold higher than the 5C-3 and 5C-AxxA viruses. Similarly, the 6I-1 virus replicated to peak levels 3-fold higher than 6I and achieved peak levels of replication 3 days earlier. Reduced replication of the 5C-AxxA virus is consistent with previous studies [54]. 5C-3 and 6I mutant viruses, which are defective for PAK2-association, but functional for CD4 and MHC Class I down modulation and infectivity enhancement, did not enhance replication compared to ΔNef virus. These results suggest that Nef-PAK2 association is important for enhancing HIV-1 replication when freshly isolated T cells are infected and sub-maximally activated.

### Nef residues important for PAK2-association are also important for enhancing T cell activation

**Figure 3:**
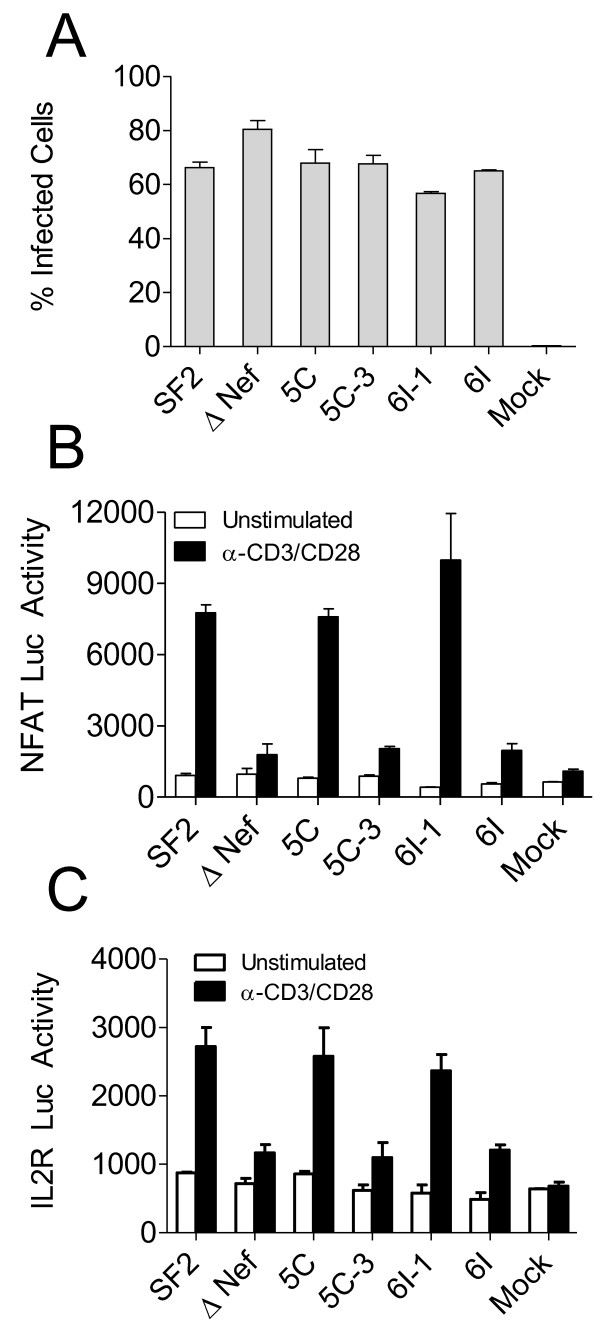
**The ability of Nef to associate with activated PAK2 correlates with the ability to enhance T cell activation**. **A**. MAGI R5+ cells were incubated for 6 h with 5,000 ^3^H cpm of RT activity of VSV-G pseudotyped virus. Infections and staining were performed as in Figure 1B. Average percent of infected (β-galactosidase+) cells from triplicate infections ± SEM are shown. **B **and **C**. 200,000 ^3^H cpm of RT activity of VSV-G pseudotyped virus were incubated overnight with 10^6 ^Jurkat cells stably expressing NFAT-Luc (**B**) or IL2R-Luc (**C**). Infected cells were then incubated with 10^6 ^α-CD3/CD28 beads for 4 h. Cells were lysed with passive lysis buffer. The lysate was freeze/thawed once and luciferase activity was assayed by luminescence. Average luciferase activity for triplicate samples ± SEM is shown.

Nef-mediated enhancement of T cell activation is a potential mechanism by which Nef may enhance viral replication [19,59]. Therefore, we sought to determine whether Nef mutants defective for PAK2-association are also defective for the ability to enhance T cell activation. Previous reports have shown that Nef expression enhances upregulation of CD25 [60] and activation of NFAT and ILR promoter elements in response to CD3 stimulation in Jurkat cells [23]. Therefore, we first examined the phenotype of Nef mutants defective for PAK2 association in Jurkat E6.1 clones stably expressing either NFAT-Luc or IL2R-Luc reporter constructs following infection with each Nef variant virus [23]. Pseudotyping with VSV-G eliminates Nef-mediated infectivity enhancement and enhances viral infectivity compared to pseudotyping with HIV Env [61]. Infection of MAGI cells with equal amounts of RT activity confirmed these prior findings. VSV-G pseudotyped viruses infected 2-fold more cells compared to viruses expressing only the HIV Env (Figure 1C vs. Figure 3A). As expected, Nef expression did not alter the ability of VSV-G pseudotyped virus to infect MAGI cells. Therefore, VSV-G pseudotyped viruses were used to allow equivalent, high-efficiency infection of the Jurkat NFAT-Luc and Jurkat IL2R-Luc reporter cells.

To determine whether Nef-PAK2 association is important for Nef-mediated enhancement of T cell activation, we infected the NFAT-Luc and IL2R-Luc reporter cells with an equal MOI of VSV-G pseudotyped virus. At 24 h post-infection, luciferase activities in unstimulated cells were equivalent between mock-infected and HIV-infected cultures irrespective of the Nef variant expressed. After 4 h of stimulation with α-CD3/CD28-coated beads, in contrast to the 72 h stimulation in Figure 2B, NFAT-Luc cells infected with SF2, 5C, and 6I-1 virus had 5-, 3.5-, and 4-fold higher levels of luciferase activity than did ΔNef, 5C-3, and 6I-1, respectively. In IL2R-Luc cells, SF2, 5C, and 6I-1 Nef had 2-, 2.5-, and 2-fold higher levels of luciferase activity than did ΔNef, 5C-3, and 6I-1, respectively (Figure 3B and 3C). After 8 h or 16 h of stimulation with α-CD3/CD28-coated beads, wild-type Nef virus did not enhance cellular activation or NFAT-luc activity compared to Nef- or mutant Nef virus unless the bead concentration was reduced (Additional File 2, Figure S2 and Additional File 3, Figure S3). Therefore, in the context of viral infection, the ability of Nef to enhance NFAT and IL2 receptor promoter-driven luciferase activity following T cell receptor stimulation correlates with its ability to associate with activated PAK2 and to enhance viral replication, and is dependent on the strength of the activating stimulus and the length of time the stimulus is applied.

### Nef residues important for PAK2-association are important for enhancing activation of primary CD4 and CD8 T cells when Nef is expressed in the absence of other viral proteins

**Figure 4:**
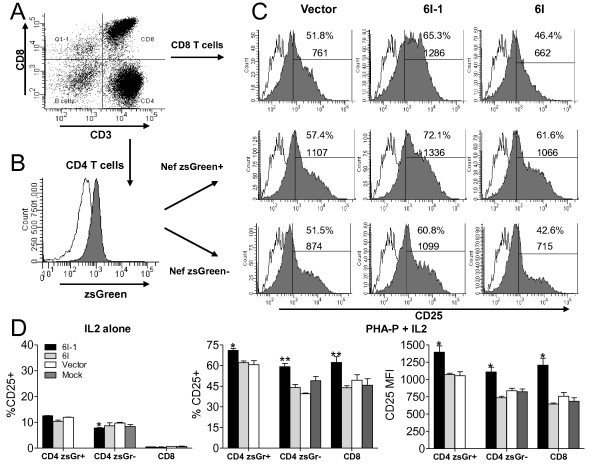
**Residues important for PAK2-association are important for enhancing T cell activation when Nef is expressed alone**. HXB2 pseudotyped lentiviral particles containing pHAGE-EF1α-Nef-IRES-zsGreen genomes were used to transduce freshly isolated PBMC. Three days post-transduction, the cells were stimulated with PHA-P (1 μg/ml) in the presence of 10 U/ml IL-2 for an additional 3 days. Replicate cultures contained 10 U/ml IL-2 alone. All cultures were then stained with α-CD3-PE-Cy5.5, CD8-PE, and CD25-APC-Cy7. Cell surface markers and zsGreen expression were monitored by flow cytometry. **A**. CD3 and CD8 expression define CD8 T cell (upper right quadrant) and CD4 T cell (lower right quadrant) populations. CD8 T cells were analyzed directly for CD25 expression. CD4 T cells were analyzed for zsGreen expression in B. **B**. zsGreen expression analysis of CD4 T cells. Transduced (Nef+ zsGreen+) and untransduced (Nef- zsGreen-) populations were defined for further analysis in C. Open histogram is the mock transduction. Shaded histogram is the sample transduced with the empty vector. **C**. CD25 expression in indicated populations. Total CD8 T cell population is shown in the upper panel. Nef+ (zsGreen+) populations are shown in the middle panel. Nef- (zsGreen-) populations are shown in the bottom panel. Percent CD25+ and CD25 MFI of the entire population is shown above the histogram. Representative plots for three samples are shown. **D**. The average % CD25 positive or CD25 MFI ± standard deviation (SD) of triplicate cultures is shown for cultures with 10 U/ml IL-2 alone (left panel) or 1 μg/ml PHA-P with 10 U/ml IL-2. *p < 0.05 ** and p < 0.005 (Student's t-test).

To determine if the correlation between Nef-PAK2 association and Nef-mediated enhancement of T cell activation (Figure 3) exists when *nef *is the only viral gene expressed, a lentiviral expression vector, pHAGE, was used to transduce *nef *genes under the control of the full EF1α promoter in PBMC. This vector contains an IRES element at the 3' end of the *nef *gene driving expression of the GFP variant zsGreen. Lentiviral vectors were packaged with HIV *gag *and *pol *and pseudotyped with the CXCR4-tropic HIV-1 envelope HXB2. At 72 h post-transduction, transduced unstimulated PBMC were incubated for 3 days with 10 U/ml IL-2 with or without 1 μg/ml PHA-P. After stimulation, cell surface CD3, CD8, CD25, and zsGreen expression was determined by FACS analysis (Figure 4A). CD4 T cells exposed to no vector (Mock) were used to set the zsGreen+ gate. 44%, 47%, and 50% of CD4 T cells were zsGreen+ when transduced by vectors expressing 6I-1 Nef, 6I Nef, and empty vector, respectively (Figure 4B). In the presence of IL-2 alone, 8-12% of CD4 T cells expressed CD25 and < 1% of CD8 T cells expressed CD25 (Figure 4D, left panel). No significant difference was observed between 6I-1 and 6I, vector, and mock in Nef+ (zsGreen+) CD4 T cells or CD8 T cells. Cultures transduced with 6I-1 Nef contained 1.2- and 1.1-fold more CD25+ Nef-(zsGreen-) CD4 T cells than cultures transduced with 6I Nef, or empty vector, respectively (p = 0.02 and 0.01). Following 3 days of 1 μg/ml PHA-P stimulation, cultures transduced with 6I-1 Nef contained 1.14- and 1.17-fold more CD25+ Nef+(zsGreen+) CD4 T cells compared to those transduced with 6I or empty vector (p = 0.005 and 0.02, respectively), 1.3-, 1.5-, and 1.2-fold more CD25+Nef-(zsGreen-) CD4 T cells compared to those transduced with 6I Nef, empty vector, or mock transduced (p = 0.002, 0.0002, and 0.01, respectively), and 1.3-, 1.4-, and 1.4-fold more CD25+ CD8 T cells compared to those transduced with 6I, empty vector or mock transduced, respectively (p = 0.002, 0.017, and 0.01). Additionally, in cultures transduced with 6I-1 Nef+(zsGreen+) CD4 T cells expressed 1.30- and 1.31-fold higher CD25 MFI compared to cultures transduced with 6I or empty vector (p = 0.025 and 0.034, respectively), CD25+Nef-(zsGreen-) CD4 T cells expressed 1.5-, 1.3- and 1.3-fold higher CD25 MFI compared to those transduced with 6I, empty vector, or mock transduced (p = 0.006, 0.024, and 0.02, respectively), and CD8 T cells expressed 1.3-, 1.4-, and 1.4-fold higher CD25 MFI compared to those transduced with 6I, empty vector, or mock transduced, respectively (p = 0.005, 0.017, and 0.01). Nef-mediated enhancement of cellular activation may have been reduced in zsGreen+ cells because lentiviral transduction likely occurred in cells that were already activated, or partially activated. After 3 days of stimulation with 1 μg/ml PHA-P in the presence of IL-2, Nef enhances cellular activation of transduced and bystander CD4 and CD8 T cells in a manner that is dependent on Nef-PAK2 association. This effect, albeit modest, is significant for two measures of T cell activation (% CD25+ and CD25 MFI) in 3 different T cell populations (Nef+ CD4+, Nef-CD4+, Nef-CD8+). Thus, Nef may increase the pool of bystander T cells permissive for replication.

### T cell activation is dependent on PAK2 both in the presence and absence of Nef

**Figure 5:**
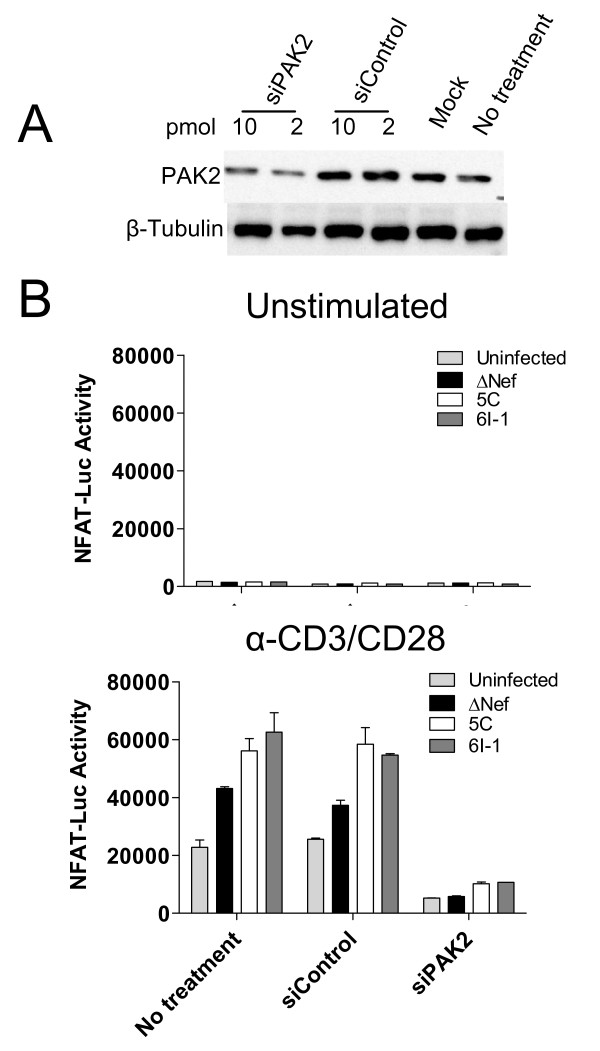
**siRNA knockdown of PAK2 in Jurkat cells reduces NFAT activation induced by anti-CD3/CD28 stimulation whether or not Nef is present**. A. Jurkat NFAT-Luc cells were transfected with 2 or 10 pmol of PAK2 siRNA or non-targeting siRNA control pool. Three days later, cells were lysed in 1% NP-40 lysis buffer and analyzed by SDS-PAGE/Western blot for PAK2 and β-tubulin expression. **B**. 200,000 ^3^H cpm of RT activity of the indicated VSV-G pseudotyped virus was incubated overnight with 10^6 ^NFAT-Luc cells transfected with 10 pmol of the indicated siRNA. Infected cells were then incubated with 10^6 ^α-CD3/CD28 beads for 4 h. Cells were lysed with passive lysis buffer. The lysate was freeze/thawed once and luciferase activity was assayed by luminescence. Average luciferase activity for triplicate samples ± SEM is shown.

To determine the requirement for PAK2 in Nef-mediated enhancement of T cell activation, we transiently transfected Jurkat NFAT-Luc cells with siRNA targeting PAK2 and reduced PAK2 expression by ~2-fold as determined by Western blotting (Figure 5A). Untransfected cells or cells transfected with 10 pmol control siRNA or PAK2 targeting siRNA were infected with VSV-G pseudotyped HIV expressing the indicated Nef as described above (Figure 3) and then stimulated 24 h post-infection with α-CD3/CD28 beads for 4 h. No difference in NFAT-Luc activity was observed between unstimulated cultures, regardless of viral infection or siRNA transfection (Figure 5B). Following α-CD3/CD28 stimulation, Jurkat cells expressing 5C or 6I-1 Nef had ~2.5 - 2.7-fold higher levels of NFAT-Luc activity compared to cells expressing the ΔNef control in cells transfected with control siRNA. Transfection with PAK2 siRNA reduced NFAT-Luc activity by 80, 85, 82, and 83% for uninfected, ΔNef, 5C, and 6I-1-expressing cells, respectively. Thus, NFAT activity in stimulated Jurkat cells is dependent on PAK2 both in the presence and absence of Nef.

**Figure 6:**
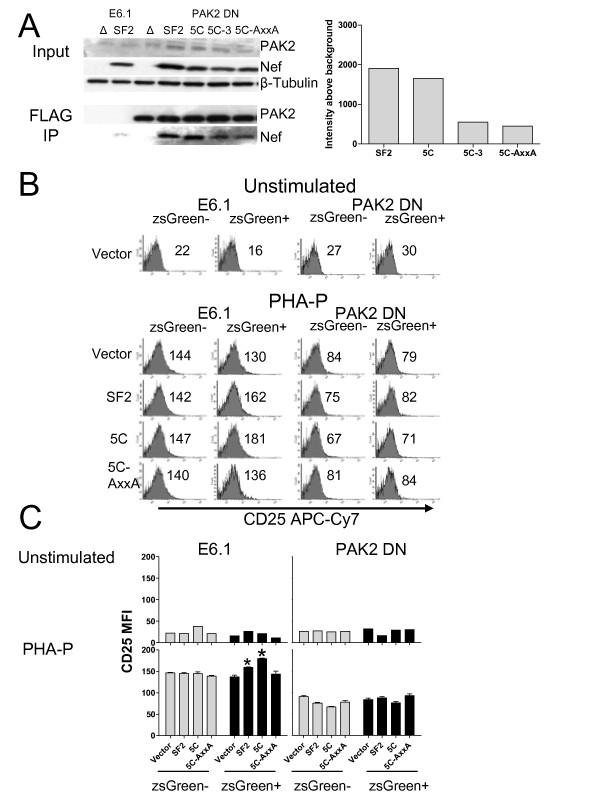
**Expression of a dominant negative PAK2 mutant inhibits Nef-mediated enhancement of CD25 expression in Jurkat cells stimulated by PHA-P (1 μg/ml)**. **A **and **B**. Jurkat E6.1 cells were transfected with pCDNA3.1 FLAG-PAK2 K278R (PAK2 DN) and passaged in 1 mg/ml G418 for 15 days. 1.5 × 10^6 ^parental E6.1 cells and PAK2 DN cells were transduced with 50,000 cpm of RT activity of the indicated pHAGE Nef-IRES-zsGreen vectors. **A**. At 48 h post-transduction, cells were lysed in 1% NP-40 lysis buffer. 10 μl of anti-FLAG agarose conjugate beads were used to immunoprecipitate FLAG-PAK2 from 1 mg of lysate. Bead bound proteins were eluted in 2X SDS sample buffer, and analyzed by SDS-PAGE/Western blot for PAK2, Nef, and β-tubulin expression. **B**. 8 h post-transduction, cells were stimulated with 1 μg/ml PHA-P for 18 h and then stained with CD25 APC-Cy7. Cell surface CD25 and zsGreen expression was determined via flow cytometry. Representative plots are labeled with CD25 MFI for zsGreen+ and zsGreen- cells. **C**. Averages of 3 replicates ± SEM CD25 MFI are shown. * Significantly different from E6.1 transduced with vector control; p < 0.01 (Student's t-test).

As a complementary approach to determine the role of PAK2 in Nef-mediated enhancement of T cell activation, we established a stable Jurkat cell line expressing FLAG-tagged PAK2 K278R (PAK2 DN), a dominant negative mutant. First, parental E6.1 cells and PAK2 DN cells were infected with Nef-bearing pHAGE-IRES-zsGreen vectors pseudotyped with VSV-G. Two days post-transduction, PAK2 K278R complexes were immunoprecipitated with agarose bound FLAG-antibodies and analyzed by SDS-PAGE/Western blot for PAK2 K278R expression and co-precipitation of Nef (Figure 6A). PAK2 K278R-FLAG expression in PAK2 DN cells was confirmed by western blotting with rabbit anti-PAK2. PAK2 K278R co-immunoprecipitated SF2 and 5C Nef, whereas co-immunoprecipitation of 5C-3 and 5C-AxxA Nef was markedly reduced compared to 5C Nef. To examine the effects of the dominant negative PAK2 mutant on Nef-mediated enhancement of T cell activation, cells were stimulated with 1 μg/ml PHA-P for 18 h, and then cell surface CD25 and zsGreen expression were determined via flow cytometry. In cells transduced with empty vector, CD25 MFI in Jurkat PAK2 DN cells was reduced by 39% compared to parental E6.1 cells (Figure 6B and 6C) (p = 0.0005), indicating that T cell activation is dependent on PAK2 in the absence of Nef. In contrast to Jurkat PAK2 DN cells, parental E6.1 cells transduced with SF2 and 5C Nef (zsGreen+) expressed 1.2- and 1.3-fold higher CD25 MFI (p = 0.0062 and 0.0005, respectively) (Figure 6B and 6C), whereas the 5C-AxxA Nef mutant had no significant effect on CD25 MFI. In Jurkat PAK2 DN cells, SF2 and 5C Nef-mediated enhancement of CD25 expression was reduced 4-fold or abolished, respectively (Figure 6B and 6C). No significant difference was observed between SF2, 5C, and 5C-AxxA Nef-expressing PAK2 DN cells compared to cells expressing the vector control (p = 0.4169, 0.1703, and 0.5304, respectively). Therefore, experiments using a dominant negative PAK2 mutant suggest that SF2 and 5C Nef-mediated enhancement of T cell activation is dependent on PAK2.

**Figure 7:**
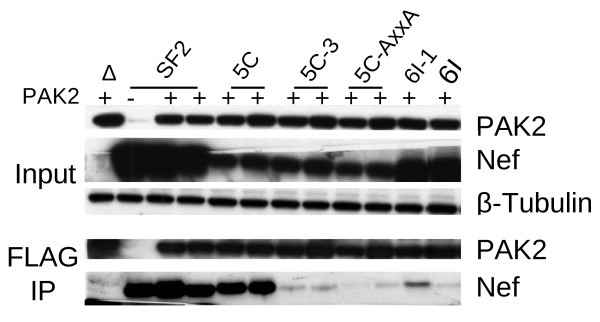
**Nef residues important for enhancement of viral replication and cell activation are also important for association of Nef with PAK2 in co-precipitation assays**. **A**. 293T cells were transfected with 0.5 μg pCR3.1 Nef-HA or empty vector, 0.5 μg pCDNA.31 FLAG-PAK2 K278R, and 0.5 μg CDC42 V12 in 6-well plates. 48 h post-transfection, cells were lysed in 1ml 1% NP-40 lysis buffer. 10 μl of anti-FLAG agarose conjugate beads were used to immunoprecipitate FLAG-PAK2 from 1 mg of lysate. Bead bound proteins were eluted in 2X SDS sample buffer, separated on a 12% polyacrylamide gel, and transferred to PVDF. Expression was detected by Western blot.

To confirm that the ability of SF2, 5C, 5C-3, 5C-AxxA, 6I-1 and 6I to associate with PAK2 correlates with the previously reported abilities of these Nefs to associate [48], or not, with activated PAK2 as determined by *in vitro *kinase assays, we transfected 293T cells with plasmids for HA-tagged Nef, CDC42 V12, and FLAG-tagged PAK2 K278R. Two days post-transfection, complexes containing PAK2 K278R were immunoprecipitated with agarose bound FLAG-antibodies and analyzed by SDS-PAGE/Western blot for co-immunoprecipitation (Figure 7). Co-immunoprecipitated Nef was normalized to the amount of input Nef (Figure 7). Despite differences in expression levels between SF2, 5C, and 6I-1 wild-type Nefs, each wild-type Nef and the corresponding mutants were expressed at similar levels. Compared to 5C Nef, the ability of the 5C-3 and 5C-AxxA mutants to associate with PAK2 was reduced (Figure 7). Compared to 6I-1 Nef, the ability of the 6I mutant to associate with PAK2 was reduced (Figure 7). Thus, in both 293T cells and Jurkat cells, the ability of wild-type and mutant Nefs to associate with PAK2 in co-precipitation assays correlates with their previously reported abilities to associate with activated PAK2 demonstrated by *in vitro *kinase activities [48] and with their ability to enhance T cell activation (Figure 3 and 4) and HIV replication (Figure 2).

## Discussion

Here, we show that the ability of Nef to associate with activated PAK2 is important for its ability to enhance HIV replication in freshly isolated T cells. Mutations at positions 89 and 191, which disrupt PAK2 association, rendered Nef defective for the ability to enhance cellular activation and viral replication in freshly isolated T cells, but not the ability to enhance viral infectivity or downmodulate CD4 and MHC Class I [48]. As expected and consistent with other reports [62,63], by targeted siRNA knockdown we show that PAK2 is important not only for Nef-mediated enhancement of T cell activation but also for activation of T cells in the absence of Nef (Figure 5). We also show that Nef-mediated enhancement of T cell activation is abrogated in the presence of a dominant negative PAK2 mutant (Figure 6). The ability of wild-type or mutant Nefs to enhance T cell activation correlated with their ability to associate with PAK2 in co-precipitation assays in Jurkat and 293T cells (Figure 6A and 7). Together, these data are consistent with a model in which enhancement of T cell activation and HIV replication occur through related mechanisms involving Nef association with PAK2, most likely within a multiprotein complex.

Previous studies suggest that Nef enhances HIV replication by reducing the threshold of cellular activation [19,24]. Consistent with this model, we found that Nef-dependent enhancement of viral replication in T cells stimulated after infection is highly dependent on the strength of activating stimuli. The greatest differences in levels of replication between Nef+ and Nef- viruses were observed in cultures sub-maximally stimulated with 1 μg/ml PHA-P for 72 h (Figure 2A). The strength of the activating stimulus correlated inversely with Nef-mediated enhancement of HIV-1 replication (Additional File 1, Figure S1). Reducing the concentration of α-CD3/CD28 beads (Additional File 2, Figure S2), or shortening the duration of stimulation (Additional File 3, Figure S3 and Figure 3, Figure 4, and [22]), is required to detect Nef-mediated enhancement of cellular activation. These findings imply that sub-maximal stimulation, regardless of whether PHA-P or α-CD3/CD28 beads are used, is required to detect Nef-mediated enhancement of viral replication and T cell activation. Excluding IL-2 from the cell culture media resulted in only 1.6% viable cells. Thus, we cannot exclude the possibility that IL-2 alone increased the number of cells permissive for infection [64]. Importantly, these findings demonstrate that Nef-mediated enhancement of replication and activation can be masked when strong stimuli are used to induce cellular activation.

Nef may enhance HIV replication via several distinct mechanisms that are not mutually exclusive. Nef enhances viral replication in freshly isolated PBMC, which contains a small fraction of activated or partially activated T cells [6,7,9]. Nef expression can enhance the promoter activity of the LTR [21] and specific host cell genes [24,59,65] via mechanisms that may involve upregulation of *tat-SF1, U1 SNRNP*, and *IRF-2 *mRNAs [59] and enhancement of NFAT activity [20,23], thereby increasing the amount of viral particles produced per cell. As we and others have demonstrated, Nef interaction with cell signaling machinery in unstimulated T cells may render them permissive for high levels of productive infection upon subsequent stimulation by reducing the threshold required for cellular activation. As such, the inverse correlation between Nef-mediated enhancement of replication and the strength of cellular stimulation (Additional File 1, Figure S1B) is likely to reflect both an increase in the number of permissive cells and increased p24 production per cell. We found that activation of bystander CD4 and CD8 T cells is enhanced in the presence of Nef expression cells. Bystander activation may be important for HIV replication in vivo, as this would be expected to increase the pool of target cells permissive for productive infection and to contribute to activation induced cell death of CD4 and CD8 T cells associated with HIV infection. Therefore, Nef-mediated enhancement of T cell activation may positively influence viral replication and pathogenesis via several distinct mechanisms.

The role of PAK2 in Nef-mediated enhancement of T cell activation is unclear because PAK2 activation is dependent on several cellular factors. The results of experiments using PAK2 siRNA knock-down and a dominant negative PAK2 mutant (Figure 5 and 6) imply that PAK2 itself, and/or molecules that interact with PAK2, are important for Nef-mediated enhancement of T cell activation. For example, activation of PAK2 is dependent on binding of CDC42-GTP to the PAK2 CRIB (Cdc42- and Rac-interactive binding) domain. CDC42 binds PAK2 only in its GTP bound state, which occurs after guanine nucleotide exchange factors (GEF), such as Vav, induce exchange of GDP for GTP. Nef may therefore interact with upstream signaling molecules such as SFKs, Vav1, or CDC42 in order to associate with activated PAK2. Identifying binding partners of Nef important for PAK2-association may help to identify other host cell factors necessary for Nef-mediated enhancement of T cell activation and viral replication.

Previous reports evaluating the contribution of Nef-PAK2 association to HIV replication reached conclusions that differ from our own. One study demonstrated that siRNA knockdown of PAK2 was not important for infection of HeLa and Jurkat cells [66]. However, freshly isolated then activated primary T cells are a more relevant cell-type for studies of Nef function. Furthermore, HeLa and Jurkat cells do not require activation by external stimuli to become permissive for HIV replication. A second study used experimental conditions that may mask Nef-mediated enhancement of T cell activation [52]. Schindler *et al*. [52] incubated Jurkat NFAT-Luc reporter cells with PHA for 16 h. However, we found that extending the time of stimulation with α-CD3/CD28 coated beads from 4 to 8 h abrogated Nef-mediated enhancement of NFAT-Luc activity (Figure 3 and Additional File 3, Figure S3) and extending the time of stimulation with 1 μg/ml PHA-P from 18 to 24 h abrogated Nef-mediated enhancement of CD25 upregulation in Jurkat E6.1 cells (Additional File 4, Figure S4). Thus, our identification of specific assay conditions in which Nef expression enhances T cell activation and HIV replication sheds light on potential explanations for different results among studies that examined the importance of PAK2 for Nef-mediated enhancement of HIV replication.

Significant controversy surrounds the issue of whether or not Nef-PAK2 association contributes to SIV pathogenesis. In two studies, rhesus macaques infected with SIV mutated in the PxxP domain of Nef did not develop high viral loads until reversion of the mutations occurred [45,67]. In contrast, one study demonstrated that SIVmac239 containing the same PxxP mutation began to develop high viral loads 4 days prior to detection of reversion to wild-type [68]. The inconsistency of these findings may relate to certain limitations of the SIV model. Because of rapid disease progression, SIVmac239 infection of macaques may not be an accurate model for the chronic phase of HIV infection. PAK2 activation may be more important during chronic infection, when immune activation is lower and infected T cells are more likely to be resting, than during acute infection and late stage disease, when T cells have higher levels of activation. Indeed, lymphoid-derived Nefs in late stages of disease acquire rare, non-conservative mutations at positions critical for PAK2-association potentially abrogating the ability of Nef to associate with PAK2 [69]. Accordingly, Nef-PAK2 association may be important for pathogenesis in vivo in the same context for which it is important for enhancing replication in vitro: when infected T cells are resting.

An important finding in our study relates to technical issues that may help to explain major discrepancies between results from different groups regarding HIV replication in freshly isolated PBMC. Three technical points were critical for reproducibility of a strong Nef-dependent phenotype in freshly isolated PBMC: 1) using PBMC isolated from fresh blood instead of cryopreserved PBMC; 2) using highly purified PBMC free of platelet contamination; and 3) using pooled human A/B serum determined to be free of endotoxin. Our preliminary studies comparing different methods for this assay suggested that PBMC derived from cryopreserved rather than fresh samples, as well as PBMC containing even low levels of contaminating platelets, are more "activated" than PBMC obtained from fresh blood or isolated free of platelet contamination. Thus, assays that use cryopreserved PBMC or are conducted in the presence of contaminating platelets are not representative of results in "resting" PBMC. Platelets can release RANTES and other soluble factors that can activate PBMC, so platelet-free PBMC preparations are critical for assays that depend on a "resting" PBMC phenotype. We observed that fetal bovine serum (FBS) enhances survival of activated PBMC compared to pooled human A/B serum, thereby increasing the number of activated cells and reducing the percentage of resting cells. Thus, stimulation in the presence of FBS may result in stronger cell stimulation than delivering the same stimulus in the presence of human A/B serum. We have shown that the ability of Nef to enhance replication in freshly isolated PBMC is highly dependent on the strength and duration of stimulation. Therefore, confounding factors that enhance the strength of stimulation may mask certain Nef phenotypes. For example, the presence of endotoxin in some serum preparations may alter the cytokine profile of PBMC. In summary, using freshly isolated rather than cryopreserved PBMC and reducing PBMC activation by factors such as platelet contamination and serum endotoxin, and determining the strength and duration of stimulation required for sub-maximal stimulation are critical in order to achieve consistent effects of Nef on HIV-1 replication in resting T cells from a given donor.

## Conclusion

HIV-1 Nef-mediated enhancement of T cell activation and viral replication in T cells is dependent on PAK2 and the strength of T cell activating stimuli, and correlates with the ability of Nef to associate with PAK2. Nef-mediated enhancement of T cell activation may increase the number of infected cells able to produce progeny virus along with the amount of virus produced per cell. Additionally, Nef increases the number of uninfected bystander cells permissive for viral replication in a manner that is dependent on Nef residues important for PAK2 association. Two structural domains appear to govern the ability of Nef to enhance activation of infected and uninfected cells: the PxxP motif, which interacts with SH3 domains, and the hydrophobic binding surface formed between residues 89 and 191, which may interact with an unknown binding partner [48]. Future analysis of the Nef-PAK2 interaction will examine the ability of Nef to associate with SH3 domain-containing cellular factors that influence both PAK2 association and T cell activation. This interaction is an attractive potential therapeutic target because an inhibitor that blocks the ability of Nef to interact with host cell factors important for enhancement of HIV-1 replication and T cell activation would be expected to reduce viral replication and, similar to infections with Nef-deleted strains of HIV and SIV, postpone the onset of AIDS.

## Materials and methods

### Proviral Construction

Primary Nefs and their mutants cloned into pCR3.1 were digested with BamHI and ClaI and inserted into the corresponding sites in a modified pNL4-3 provirus [70]. SF2 Nef was inserted via MluI and ClaI sites. Nef-negative pNL4-3 ΔXhoI was provided by Damian Purcell. Viral stocks were produced by CaPO_4 _transfection of 3 × 10^6 ^293T cells in a 10 cm plate with 10 ug of provirus and, where indicated, 1 ug of pVSV-G. Viral stocks were assayed for reverse transcriptase activity as described previously [71].

### Lentiviral Vector Construction and Production

pCR3.1 Nef plasmids were amplified with primers 5'AAAAGCGGCCGCCACCATGGGTGGCAAGTGGTCAAAA3' and 5'AAGGATCCTCATGAAGCGTAATCTGGCAC 3', which add a NotI site and a Kozak sequence to the 5' end of Nef and a BamHI site to the 3' end of Nef. Nef genes were inserted into pHAGE fEF1α-IRES-zsGreen which was kindly provided by the Harvard Gene Therapy Initiative [72]. 3.5 μg of pHAGE vector,3.5 μg of the packaging construct pDR89.1 [73], and 1 μg of pVSV-G were transfected by lipid transfection (LipoD293T, Signagen) into 5.5 × 10^6 ^293T cells plated 24 h prior in a 10-cm tissue culture plate. Medium was changed at 18 h and vector containing supernatants were harvested after an additional 24 hours. Vector containing supernatants were assayed for RT activity as described previously [71].

### PBMC Isolation and Infection

Healthy HIV-/HCV- donor blood provided by Research Blood Components (Boston, MA) was collected into EDTA vacutainer tubes. The protocol for obtaining blood was approved by the Dana-Farber Cancer Institute IRB. After collection, 10 mls of fresh blood were diluted 1:1 with PBS and layered over 15 ml Ficoll Histopaque 1077 (Sigma) and spun at 2000 × g for 20 min at room temperature. 5 mls of PBMC containing serum were collected at the interface and diluted with 20 mls of PBS. The diluted cells were pelleted at 1000 × g for 10 min at 4°C, resuspended in 10 mls of PBS, and pelleted again at 1000 × g for 10 min. The cell pellet was resuspended in 10 mls of RPMI-1640 (Invitrogen) and pelleted at 1000 × g for 10 min. Cells were counted and resuspended at 2 × 10^6^/ml in RPMI-1640 with 10% pooled Human A/B sera (Gemini) and 5 U/ml penicillin/streptomycin. Infections contained 8 X10^5 ^cells in 800 μl with 4,000 ^3^H cpm of RT activity for SF2, 5C, 5C-3, 6I-1, and 6I Nef-bearing proviruses or 12,000 and 16,000 ^3^H cpm of RT activity for pNL4-3 ΔXhoI and 5C-AxxA, respectively. Freshly-isolated PBMC were incubated overnight in a 15 ml conical tube at 37°C and washed twice in PBS the following day. Cells were resuspended in 800 μl of media and incubated for 2 days. On day 3 post-infection, cells were pelleted and resuspended in media containing 10 u/ml IL-2 with either 1 ug/ml PHA-P (Sigma), 2 ug/ml PHA-P, or α-CD3/CD28 beads (Invitrogen) at a bead-to-cell ratio of 1:1. Three days post-activation (day 6 post-infection), infected cultures were washed twice and returned to media containing 10 u/ml IL-2. Replicate samples of activated PBMC were analyzed for levels of cell activation as described below. Two hundred μl of resuspended cultures were plated in triplicate in a 96-well U-bottom plate. Every three or four days, 0.5 volume of media was removed and saved, and replaced with 0.5 volume fresh media to compensate for evaporation. Viral replication was measured by p24 ELISA (Perkin-Elmer) of culture supernatants.

### FACS analysis

2 × 10^5 ^PBMC were washed twice with cold PBS containing 2 mM EDTA (PBS-E) and resuspended in 200 μl of PBS-E containing 2% FBS with 5 μl of CD3-FITC (BD Pharmingen) and 5 μl of CD25-PE (BD Pharmingen). Cells and antibody were incubated at 4°C for 45 min. Cells were washed twice with PBS-E and resuspended in PBS and analyzed on a BD FACSCanto II cell cytometer. FACS analysis was done using BD FACSDiva software.

### MAGI Infection

4 × 10^4 ^MAGI indicator cells (NIH AIDS Reagent Repository) were plated in a 12-well plate in selection media (Dulbecco's modified Eagle's medium (DMEM; Invitrogen) supplemented with 10% fetal calf serum, 5 U/ml pen/strep plus 0.2 mg/ml G418 (at an active concentration of 700 μg per mg), 50 U/ml hygromycin, and 1 μg/ml puromycin. Twenty-four hours later, selective media was removed from three wells at a time and replaced with 5000 cpm of RT activity in 300 μl of DMEM plus 20 μg/ml of DEAE-Dextran. Cells and virus were incubated for 6 h at 37°C. Cells were washed three times with PBS and incubated for 36 h at 37°C in 1 ml of DMEM without selection. Cells were washed once and fixed with 1 ml of 1% formaldehyde, 0.2% glutaraldehyde in PBS for 5 min. The cells were washed twice with PBS and incubated with PBS containing 4 mM potassium ferrocyanide, 4 mM potassium fericcyanide, 2 mM Mg_2_Cl, and 0.4 mg/ml X-gal for 50 min at 37°C in a dry incubator. Three random fields from each of three triplicate wells were counted for each infection. Percent Infected cells = β-galactosidase + cells (blue cells) in field/total number of cells in field × 100%.

### Luciferase Reporter Assay

IL2R-Luc and NFAT-Luc Jurkat E6.1 cells were kindly provided by Dr. Michel Tremblay, University of Laval, Montreal, Canada [23]. 5 × 10^5 ^cells were plated in 1 ml of non-selective media with 200,000 cpm of RT of VSV-G pseudotyped vectors and incubated for 18 h. Cells were washed twice with PBS and resuspended in 1 ml of media containing 5 × 10^5 ^α-CD3/CD28 beads (Invitrogen). After 6 h of incubation at 37°C, the cells were pelleted, washed twice with PBS and resuspended in 200 μl Passive Lysis Buffer (Stratagene), incubated at room temperature for 15 min, and subjected to one round of freeze/thaw. Twenty μl of lysate was added to wells of a 96-well white-wall black plate. 100 μl Stratagene Luciferase Assay buffer mixture was added and luciferase activity was read on a Bethold Centro LB 960 luminometer.

### Lentiviral transduction

2 × 10^6 ^freshly-isolated PBMC were incubated in 1 ml of media containing 750,000 ^3^H cpm of RT activity in the presence of 8 μg/ml polybrene. Cells were spun for 2 h at 2,000 RPM at 30°C, incubated overnight. Media was removed and 10% glycerol in PBS solution was added and immediately washed twice with PBS. 2 × 10^5 ^cells were added to a U-bottom, 96-well plate in media containing 10 U/ml IL-2 for three days. Fresh media containing 1 μg/ml PHA-P and 10 U/ml IL-2 or IL-2 alone were added to the cultures and incubated for 72 h. Cells were then washed twice with cold PBS-E and resuspended in 200 μl PBS-E FBS containing 1 μl of CD25-APC-Cy7 (BD Pharmingen), 5 μl CD8-PE, and 5 μl CD3-PE Cy5.5. Cells were incubated for 45 min at 4°C, washed twice with PBS-E, and resuspended in PBS. Acquisition and analysis were done with a BD FACSCanto cell cytometer using BD FACSDiva software. FSC and SSC were used to define a lymphocyte population consistent between all samples.

### siRNA transfection of Jurkat E6.1 cells

5 × 10^6 ^Jurkat E6.1 cells were nucleofected in 100 μl AMAXA Solution V in program S-18 with 10 or 2 pmol of siRNA targeting PAK2 (SmartPool, Dharmacon) or non-targeting control siRNA (Dharmacon). Cells were cultured for 48 h in 10% FBS RPMI supplemented with 1X GlutaMax (Invitrogen), prior to infection or 66 h prior to SDS-PAGE/Western blot analysis for PAK2 expression. SDS-PAGE/Western blotting was carried out as described above. Blots were probed with anti-β-tubulin (1:1000, Sigma-Aldrich) and rabbit anti-PAK2 (1:1000, Cell Signaling).

### Generation of Jurkat cells stably expressing a dominant negative PAK2 mutant

10^6 ^Jurkat E6.1 cells were nucleofected in 100 μl AMAXA Solution V in program S-18 with 1 μg of pCDNA3.1FLAG-PAK2 K278R. Cells were passaged in 1 mg/ml G418 10% FBS RPMI supplemented with 1X GlutaMax (Invitrogen) for 15 days. Expression of the FLAG-tagged PAK2 mutant was confirmed by Western blot with anti-FLAG. 3 × 10^6 ^parental control or PAK2 DN-expressing Jurkat E6.1 cells were transduced with 25,000 cpm of RT activity of pHAGE-Nef-IRES-zsGreen vectors. 8 h post-transduction, cells were washed twice and stimulated with 1 μg/ml PHA-P for 18 h, following which cells were stained with CD25 APC-Cy7 and analyzed by flow cytometry for zsGreen and CD25 expression.

### Nef-PAK2 co-immunoprecipitation in Jurkat E6.1 PAK2 DN cells

3 × 10^6 ^Jurkat E6.1 parental or Jurkat E6.1 PAK2 DN cells were transduced with 600,000 cpm of RT activity of VSV-G pseudotyped pHAGE Nef-IRES-zsGreen vectors. Cells were incubated for 16 h with lentiviral vectors and then washed twice with PBS. After 48 h in culture, cells were washed twice with PBS and lysed in 1 ml 1% NP-40 lysis buffer. Immunocomplexes were isolated and analyzed as described below.

### Nef-PAK2 co-immunoprecipitation in 293T cells

4.5 × 10^5 ^293T cells were seeded into 6-well plates 24 h prior to transfection. 0.5 μg pCR3.1 Nef-HA or empty vector, 0.5 μg pCDNA.31 FLAG-PAK2 K278R, and 0.5 μg CDC42 V12 in 75 μl serum-free DMEM were mixed with 3 ul GenJet (SignaGen) in 75 μl serum-free DMEM, incubated for 15 minutes, and added to culture wells. 48 h post-transfection, cells were washed twice with PBS and lysed in 1 ml 1% NP-40, 50 mM Tris, 150 mM NaCl, pH 8.0 lysis buffer containing Compete Protease Inhibitor (Roche) and PhosphoStop phosphatase inhibitor (Roche). Insoluble material was pelleted by centrifugation at 14,000 × g for 10 min at 4°C. Protein concentration was determined by DC Assay (BioRad) and samples were diluted to 1 mg/ml in lysis buffer. Samples were precleared with 5 μl Protein A/G Plus beads (SCBT) for 1 h. FLAG-tagged PAK2 was immunoprecipitated by incubating precleared samples with 10 μl anti-FLAG M2 agarose (Sigma-Aldrich) for 16 h at 4°C. Beads were pelleted at 5000 × g for 30 seconds and washed 4X with lysis buffer. Bound protein was eluted by boiling beads with 2X SDS sample buffer. Samples were subjected to SDS-PAGE (12% polyacrylamide) and transferred to PVDF for Western blotting. Blots were blocked with 5% milk in TBS + 0.05% Tween-20, and then probed with HA-HRP (1:500, Roche), FLAG-HRP (1:500, Sigma), anti-β-tubulin (1:1000, Sigma-Aldrich) and anti-CDC42 (SCBT, 1:200).

## Competing interests

The authors declare that they have no competing interests.

## Authors' contributions

KCO, and DG designed research; KCO and JM performed research; KCO and DG, analyzed data; and KCO and DG wrote the paper. JM edited the paper. All authors read and approved the final paper.

## Supplementary Material

Additional file 1 **Figure S1. CD25 Median FI (MFI) correlates positively with peak p24 concentration and inversely with Nef-mediated enhancement of viral replication**. **A**. Correlation between peak p24 concentrations from SF2 virus replication and CD25 MFI of the CD3+ population in cultures stimulated with 1 μg/ml PHA-P, 2 μg/ml PHA-P, and αCD3/CD28-coated beads. **B**. Correlation between (Peak p24 concentration of SF2 virus - peak p24 concentration of Nef)/peak p24 concentration of SF2 virus replication versus CD25 MFI of the CD3+ population. Spearman correlation was performed using GraphPad Prism. Click here for file

Additional file 2 **Figure S2. Reducing α-CD3/CD28-coated bead concentration enhances Nef-mediated enhancement of T cell activation**. 50,000 ^3^H cpm of RT activity of VSV-G pseudotyped pHAGE- IRES zsGreen vectors was incubated overnight with 2.5 × 10^6 ^Jurkat E6.1 cells. 18 h post-transduction 2 × 10^5 ^transduced cells were stimulated with the indicated concentration of α-CD3/CD28-coated beads or left unstimulated for 16 h in one well of a 96-well U-bottom plate. Cells were then stained for CD25. CD25 and zsGreen expression were determined by flow cytometry. %CD25+ of the zsGreen population is reported for duplicate cultures. Average %CD25+ for duplicate samples ± SEM is shown. *p < 0.05 and ** p < 0.005 (Student's t-test). Click here for file

Additional file 3 **Figure S3. Nef-mediated enhancement of activation is not detected after 8 hours stimulation with α-CD3/CD28 beads**. Experiments were carried out as in Figure 3. 200,000 ^3^H cpm of RT activity of VSV-G pseudotyped virus was incubated overnight with 10^6 ^Jurkat cells stably expressing NFAT-Luc. Infected cells were then incubated with 10^6 ^α-CD3/CD28 beads for 8 h. Cells were lysed with 500 μl passive lysis buffer. The lysate was freeze/thawed once and luciferase activity was assayed by luminescence. Average luciferase activity for duplicate samples ± SEM is shown. Click here for file

Additional file 4 **Figure S4. Nef-mediated enhancement of activation is not detected after 18 hours stimulation with 1 μg/ml PHA-P**. 50,000 ^3^H cpm of RT activity of VSV-G pseudotyped pHAGE- IRES zsGreen vectors (wild-type 5C, PAK2-association defective mutant 5C-7 (F89H, H191F), or empty Vector) was incubated overnight with 2.5 × 10^6 ^Jurkat E6.1 cells. 18 h post-transduction 2 × 10^5 ^transduced cells were stimulated with 1 μg/ml PHA-P for 18 or 24 h or left unstimulated for 18 h in one well of a 96-well U-bottom plate. Cells were then stained for CD25. CD25 and zsGreen expression were determined by flow cytometry. %CD25+ of the zsGreen population is reported for duplicate cultures. Average %CD25+ for triplicate samples ± SEM is shown. *p < 0.05 and ** p < 0.005 (Student's t-test). Click here for file
