# Supplementary figures and images for: Nef-mediated enhancement of cellular activation and human immunodeficiency virus type 1 replication in primary T cells is dependent on association with p21-activated kinase 2
Source: Retrovirology. 2011 Aug 5;8:64. doi: 10.1186/1742-4690-8-64 (PMC3169461; doi:10.1186/1742-4690-8-64)

A

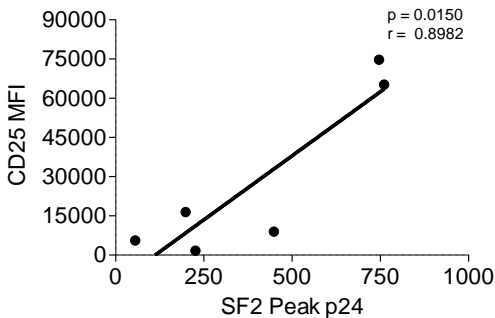

B

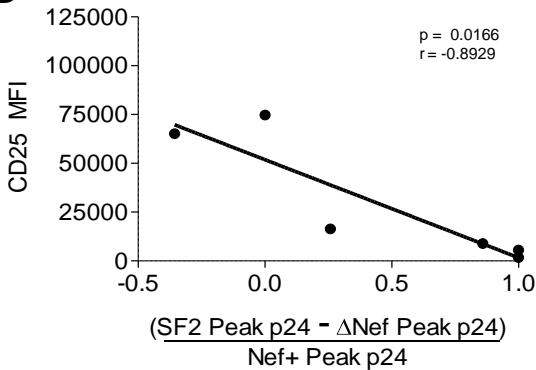

Supplement: Additional file 1 — Figure S1. CD25 Median FI (MFI) correlates positively with peak p24 concentration and inversely with Nef-mediated enhancement of viral replication. A. Correlation between peak p24 concentrations from SF2 virus replication and CD25 MFI of the CD3+ population in cultures stimulated with 1 μg/ml PHA-P, 2 μg/ml PHA-P, and αCD3/CD28-coated beads. B. Correlation between (Peak p24 concentration of SF2 virus - peak p24 concentration of Nef)/peak p24 concentration of SF2 virus replication versus CD25 MFI of the CD3+ population. Spearman correlation was performed using GraphPad Prism. [file 1742-4690-8-64-S1.PDF]

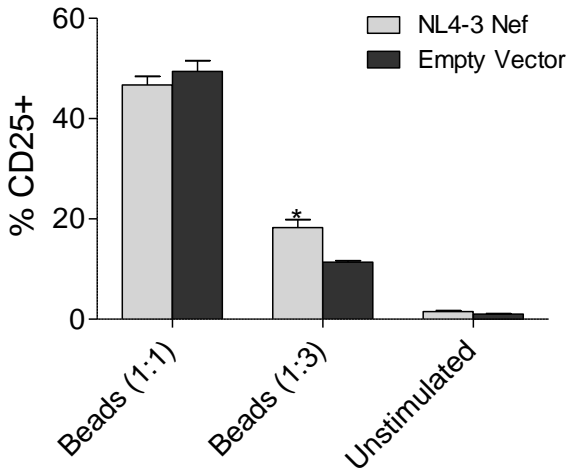

Supplement: Additional file 2 — Figure S2. Reducing α-CD3/CD28-coated bead concentration enhances Nef-mediated enhancement of T cell activation. 50,000 3H cpm of RT activity of VSV-G pseudotyped pHAGE- IRES zsGreen vectors was incubated overnight with 2.5 × 10^6^ Jurkat E6.1 cells. 18 h post-transduction 2 × 10^5^ transduced cells were stimulated with the indicated concentration of α-CD3/CD28-coated beads or left unstimulated for 16 h in one well of a 96-well U-bottom plate. Cells were then stained for CD25. CD25 and zsGreen expression were determined by flow cytometry. %CD25+ of the zsGreen population is reported for duplicate cultures. Average %CD25+ for duplicate samples ± SEM is shown. *p < 0.05 and ** p < 0.005 (Student's t-test). [file 1742-4690-8-64-S2.PDF]

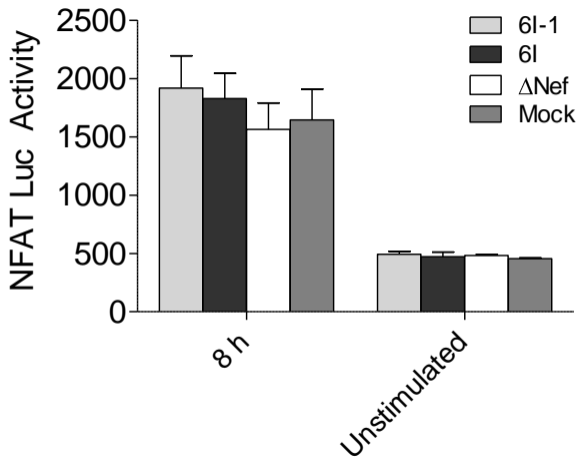

Supplement: Additional file 3 — Figure S3. Nef-mediated enhancement of activation is not detected after 8 hours stimulation with α-CD3/CD28 beads. Experiments were carried out as in Figure 3. 200,000 3H cpm of RT activity of VSV-G pseudotyped virus was incubated overnight with 106 Jurkat cells stably expressing NFAT-Luc. Infected cells were then incubated with 106 α-CD3/CD28 beads for 8 h. Cells were lysed with 500 μl passive lysis buffer. The lysate was freeze/thawed once and luciferase activity was assayed by luminescence. Average luciferase activity for duplicate samples ± SEM is shown. [file 1742-4690-8-64-S3.PDF]

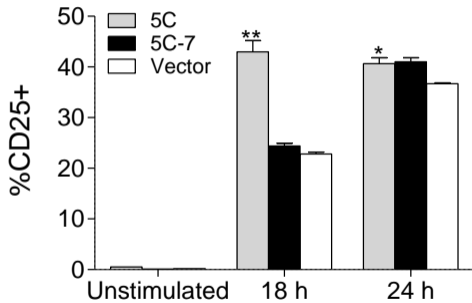

Supplement: Additional file 4 — Figure S4. Nef-mediated enhancement of activation is not detected after 18 hours stimulation with 1 μg/ml PHA-P. 50,000 3H cpm of RT activity of VSV-G pseudotyped pHAGE- IRES zsGreen vectors (wild-type 5C, PAK2-association defective mutant 5C-7 (F89H, H191F), or empty Vector) was incubated overnight with 2.5 × 10^6^ Jurkat E6.1 cells. 18 h post-transduction 2 × 10^5^ transduced cells were stimulated with 1 μg/ml PHA-P for 18 or 24 h or left unstimulated for 18 h in one well of a 96-well U-bottom plate. Cells were then stained for CD25. CD25 and zsGreen expression were determined by flow cytometry. %CD25+ of the zsGreen population is reported for duplicate cultures. Average %CD25+ for triplicate samples ± SEM is shown. *p < 0.05 and ** p < 0.005 (Student's t-test). [file 1742-4690-8-64-S4.PDF]
